# Will It Come to This?

**Published:** 1897-04

**Authors:** 


### EDITORIAL.

The office of the Business Manager of THE JOURNAL is 311 and 312 Fitten Building.

The editors are not responsible for views expressed by contributors.

Articles for publication should reach this office not later than the 15th inst.

Address all communications, and make all remittances payable to THE ATLANTA MEDICAL AND SURGICAL JOURNAL, Atlanta, Ga.

Reprints of articles will be furnished, when desired, at a small cost. Requests for the same should always be made *on the manuscript*.

### WILL IT COME TO THIS?

Reputable practitioners who are unwilling to abandon the old ethical landmarks have cause to view with some alarm certain medical innovations which now threaten to come to the front. The establishment in some of our large cities of Medical Relief Societies and Hospital Ticket Associations, and the encouragement of these by regular practitioners of medicine cannot be regarded as a very wholesome and auspicious indication as to the future state of medical practice. Some journals and some conservative individuals are asking, "where we are at"; others have taken fright and are saying that the times are out of joint, and that the melancholy days have at last come.

One of these stock medical companies has just been organized in New York. Extracts from its prospectus are as follows (*Medical News*):

"THE MEDICAL TREATMENT COMPANY.

INCORPORATED UNDER THE LAWS OF THE STATE OF NEW YORK.

OFFICE: 19 PARK PLACE, NEW YORK CITY.

"For $1 a month the Medical Treatment Company provides a doctor as often as you need one, who will treat you or your family at his office, or at your home, as the severity of the case requires, and has the prescription filled at the druggist's without extra cost to you. . . . The medical staff is composed of physicians who are graduates of the leading medical colleges of this State, and are in every way fully qualified to treat any case of disease. . . The birth of a child is a physiological condition, and therefore not classed under medical treatment. Under our system, upon payment of $5, at the time of the birth, to the attending physician, the mother will receive proper attention during convalescence."

There are already three of these institutions in St. Louis. Here are extracts from the circular of one (*American Journal of Surgery and Gynecology*):

ST. LOUIS HOSPITAL TICKET ASSOCIATION.

"This association is no longer an experiment, but a prosperous institution. It appeals to you, reader, and asks you: Have you any money laid by in case of sickness or accident, and if so, are you prepared to pay old war prices for surgery and medicine, and if you have no money saved will you throw yourself into the cold arms of charity after reading our proposition?

"*Our Contract.*—This association contracts to give you the advice of eminent and able physicians and surgeons when needed—all the medicine you require for any ailment you may have—and when you get sick or meet with an accident admit you to a first-class hospital, where you will be more humanely and tenderly cared for than you could be at home by your best friends. The association agrees, in addition to medical advice, surgery and medicine, to provide you with bed, board, and educated and trained nurses, who will give you the most tender and careful attention, and that the medicine prescribed by the physician is given just when and as directed by him, and render all those little attentions so necessary to make a sick or injured man comfortable and get him well.

"The patient is surrounded by all the modern facilities for use in case of sickness or accident; a complete register is kept of his temperature, respiration, action of medicine, and of what he eats and its effect; besides, the physician is always there and knows when the critical period comes and passes and takes advantage of it. Members' families, in case of sickness, may apply at hospital for treatment without charge except for cost of medicine.

"*Dues, Etc.*—You are offered all these advantages for the small sum of fifty (50) cents per month, with the assurance that there is positively no extra charge for anything you may require—and they are advantages such as you could not have at home without costing you a large sum of money.

"The monthly dues of fifty cents must be paid in advance. Any member whose dues are paid may apply at the office of the association for medical advice or treatment at any time, and, if seriously sick, will be sent for and admitted to the hopital."

Of course Chicago will not be outdone in such enterprise. There is at least one of these concerns there.

Unfortunately the names of several well-known physicians, surgeons, and special practitioners appear upon the staffs of these companies. The profession, the better part of it, at least, should promptly stamp its positive disapproval of such associations and should at once serve notice upon those doctors affiliating with them that they do so at the price of their professional standing. It is devoutly to be hoped that these concerns will have the life knocked out of them in the places of their birth and that none will show their heads elsewhere. The *Medical News* in a good editorial says:

"If a rest from his labors is what the doctor longs for, verily his millennium is at hand. Hospitals, clinics, and dispensaries have long since considerably lightened his labors, but it remained for a private company to put him completely at his ease by providing for patients too lazy or proud to go to dispensaries. The general practitioner has for some time been in doubt as to whether the public really needed him, but now all doubt has been cast aside, and he can get a job in the woodyard of the Charity Organization Society or retire to the almshouse, from whose tranquil precincts he can watch the death-throes of legitimate medicine.

"To the unthinking the workings of this company may appear somewhat revolutionary, but to one who has watched the signs of the times it seems only the legitimate outgrowth of the commercial spirit which has invaded and is likely to engulf medicine. That such a company may receive patronage enough to insure financial success is possible, and that it can hire regular physicians to do its work is probable. The extent to which the public will go for "bargains" has no limit, and the willingness with which doctors will do contract work is well known.

"Surely the (downward) path of the doctor is being made easy, and he may soon say with Solomon: 'What profit hath he that worketh in that wherein he laboreth?'"

